# Efeitos de Longo Prazo do Implante Valvar Pulmonar e Evolução da Prótese em Pacientes com Tetralogia de Fallot Corrigida

**DOI:** 10.36660/abc.20230585

**Published:** 2024-07-10

**Authors:** Luiz Fernando Caneo, Aida Luiza Ribeiro Turquetto, Matheus Negri Boschiero, Luciana Patrick Amato, Walther Yoshiharu Ishikawa, Fabiana Padilha Hodas, Melissa Ganeko Ligeiro, Daniela Regina Agostinho, Leonardo Augusto Miana, Carla Tanamati, Rilvani Cavalcante Gonçalves, Juliano Gomes Penha, Maria Raquel Brigoni Massoti, Marcelo Biscegli Jatene, Fabio Biscegli Jatene

**Affiliations:** 1 Instituto do Coração Hospital das Clínicas da Faculdade de Medicina Universidade de São Paulo São Paulo SP Brasil Instituto do Coração do Hospital das Clínicas da Faculdade de Medicina da Universidade de São Paulo, São Paulo, SP – Brasil; 2 Universidade São Francisco Faculdade de Medicina Bragança Paulista SP Brasil Universidade São Francisco Faculdade de Medicina, Bragança Paulista, SP – Brasil

**Keywords:** Tetralogia de Fallot, Função Ventricular Direita, Biomarcadores, Estratificação de Risco Genético

## Abstract

**Fundamento:**

A regurgitação valvar pulmonar é uma importante complicação de longo prazo em pacientes com tetralogia de Fallot (TF).

**Objetivo:**

O presente estudo tem como objetivo investigar os efeitos do implante valvar pulmonar (IVP) na anatomia e função do ventrículo direito (VD) e na evolução em longo prazo da prótese implantada em posição pulmonar.

**Métodos:**

Uma análise de coorte retrospectiva e unicêntrica foi realizada em 56 pacientes consecutivos com TF submetidos a IVP. O estudo incluiu pacientes de ambos os gêneros, com idade ≥ 12 anos e compreendeu avaliação de dados clínicos e cirúrgicos, ressonância magnética cardiovascular pré e pós-operatória e dados ecocardiográficos obtidos mais de 1 ano após IVP.

**Resultados:**

Após o IVP, houve uma diminuição significativa do volume sistólico final do VD indexado pela área de superfície corpórea (ASC), de 89 mL/ASC para 69 mL/ASC (p < 0,001) e do volume diastólico final indexado do VD, de 157 mL/ASC para 116 mL/ASC (p < 0,001). Além disso, houve aumento da fração de ejeção corrigida do VD [FEVDc = fluxo pulmonar ajustado (fluxo pulmonar anterógrado − fluxo regurgitante) / volume diastólico final do VD] de 23% para 35% (p < 0,001) e da fração de ejeção do ventrículo esquerdo de 58% para 60% (p = 0,008).] de 23% para 35% (p < 0,001) e da fração de ejeção do ventrículo esquerdo de 58% para 60% (p = 0,008). No entanto, foi observado um aumento progressivo no gradiente de pico da válvula pulmonar ao longo do tempo, com 25% dos pacientes apresentando um gradiente superior a 60 mmHg. Próteses menores (tamanhos 19 a 23) foram associadas a um risco 4,3 vezes maior de gradiente > 60 mmHg em comparação com próteses maiores (tamanhos 25 a 27; p = 0,029; intervalo de confiança: 1,18 a 17,8).

**Conclusão:**

Conforme esperado, o IVP demonstrou melhorias nos volumes e na função do VD. O acompanhamento e a vigilância a longo prazo são cruciais para avaliar a durabilidade da prótese e detectar potenciais complicações. O dimensionamento adequado das próteses é essencial para melhorar a longevidade da prótese.

## Graphical abstract

**Figura Central:**
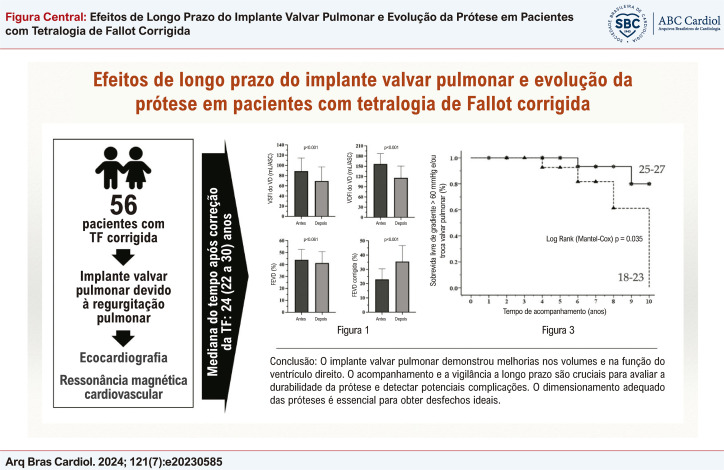
: Efeitos de Longo Prazo do Implante Valvar Pulmonar e Evolução da Prótese em Pacientes com Tetralogia de Fallot Corrigida

**Figura Central:** : Efeitos de Longo Prazo do Implante Valvar Pulmonar e Evolução da Prótese em Pacientes com Tetralogia de Fallot Corrigida

*TF: tetralogia de Fallot.*

## Introdução

A regurgitação valvar pulmonar é uma complicação comum de longo prazo observada em pacientes submetidos à correção cirúrgica da tetralogia de Fallot (TF). Essa condição leva a efeitos deletérios, como aumento progressivo dos volumes do ventrículo direito (VD), comprometimento da função ventricular, ritmos cardíacos irregulares, insuficiência cardíaca, redução da capacidade de exercício e risco elevado de morte súbita e prematura.^1,2^

O implante valvar pulmonar (IVP) surgiu como uma abordagem de tratamento promissora para resolver esses problemas. No entanto, determinar o momento ideal para o IVP exige uma avaliação de risco precisa.^3^ É importante notar que, mesmo com as melhorias iniciais no tamanho e função do VD após o IVP, a válvula implantada ainda pode deteriorar-se ao longo do tempo.^4^ O momento ideal para o IVP continua a ser um desafio, pois é necessário haver um equilíbrio entre uma intervenção demasiadamente precoce e os riscos de complicações, como endocardite ou necessidade de reintervenção para novo IVP, devido à durabilidade limitada das próteses valvares. Por outro lado, intervir demasiadamente tarde pode limitar a recuperação funcional do VD, levando a arritmias de início tardio e insuficiência cardíaca.^5,6^ Além disso, independentemente do tamanho da prótese, as taxas de reintervenção variam de acordo com o tipo de válvula implantada.^7^ A ausência de uniformidade nos protocolos de seleção de válvulas dentro e entre unidades de saúde, motivada pela preferência do cirurgião ou pelo período cronológico da implantação, interfere na análise sistemática dos desfechos publicados.^8,9^ Diferentemente dos países desenvolvidos, nem todas as próteses utilizadas comercialmente nos estudos estão disponíveis no mercado nacional, principalmente no setor de saúde pública, devido aos seus custos elevados.

O presente estudo teve como objetivo investigar os efeitos do IVP na anatomia e função do VD e na evolução em longo prazo da prótese implantada na posição pulmonar.

## Métodos

Trata-se de um estudo de coorte retrospectivo e unicêntrico realizado em 56 pacientes consecutivos no pós-operatório tardio de correção de TF submetidos a IVP devido a regurgitação pulmonar moderada a grave entre 2011 e 2018 em nossa instituição (Figura Central). O Comitê de Ética em Pesquisa com Seres Humanos institucional aprovou o estudo sob CAAE 50224615.7.0000.0068.

### Critérios de inclusão

O presente estudo incluiu pacientes que foram submetidos à correção de TF e posteriormente receberam IVP quando sintomáticos ou aqueles assintomáticos com regurgitação pulmonar grave com pelo menos um dos seguintes critérios: redução objetiva progressiva na capacidade de exercício, dilatação progressiva do VD, disfunção sistólica progressiva do VD, regurgitação tricúspide (pelo menos moderada) ou arritmias atriais/ventriculares sustentadas.^10,11^ Recomendações individualizadas para IVP foram feitas após revisões multidisciplinares. Foi necessário o consentimento informado dos pacientes para participar do estudo. Foram incluídos pacientes de ambos os sexos, com idade igual ou superior a 12 anos, com tempo de acompanhamento superior a 1 ano após IVP, que aceitaram participar do estudo e assinaram o termo de consentimento informado.

### Detalhes cirúrgicos

A cirurgia foi realizada com circulação extracorpórea e coração batendo quando viável. Um remendo transanular foi utilizado em 55% dos pacientes para corrigir a TF, que foi trocado ou adicionado em 81% durante o IVP devido à calcificação do remendo de pericárdio bovino original. Uma prótese de pericárdio bovino de fabricação nacional (Braile Biomédica, São Paulo, Brasil) foi implantada em todos os pacientes, com tamanho variando de 19 a 27 mm.

### Avaliação de imagem

Coletamos e analisamos dados obtidos de ressonância magnética cardiovascular (RMC) e ecocardiografia. Os exames de RMC foram realizados em 2 momentos: antes da cirurgia (pré-operatório) e mais de 1 ano após o IVP. O objetivo da RMC foi avaliar o volume sistólico final indexado (VSFI) do VD, o volume diastólico final indexado (VDFI) do VD, a fração de ejeção do ventrículo direito (FEVD) e a fração de ejeção do ventrículo esquerdo (FEVE). Utilizamos curvas de Kaplan-Meier para analisar a sobrevida livre de gradiente de pico superior a 60 mmHg e/ou indicação de troca valvar pulmonar de acordo com o tamanho da prótese utilizada na cirurgia. O tempo de acompanhamento foi calculado utilizando a data do último ecocardiograma obtido no acompanhamento pós-operatório ou antes da troca valvar pulmonar subtraída da data do IVP.

### Ressonância magnética cardiovascular

Todos os pacientes foram submetidos a exames de RMC em um sistema de ressonância magnética (RM) 1,5T (Philips Achieva scanner, Holanda). A função ventricular foi avaliada por cine-RM com sequência SSFP (*steady-state free precession*). Os volumes e a função ventricular foram medidos pelo método de Simpson em imagens de eixo curto e indexados pela área de superfície corpórea. O volume sistólico foi calculado deduzindo o VSFI do VDFI. O fluxo pulmonar e a regurgitação foram medidos com sequências de contraste de fase. A angiografia por RM com contraste foi usada para visualizar a árvore vascular pulmonar e suas conexões, e o realce tardio com gadolínio foi usado para detectar fibrose miocárdica (pela técnica de limiarização).^12^ A fração regurgitante pulmonar foi calculada a partir do mapa da velocidade de fase em um plano que corta o tronco pulmonar principal. A fração de ejeção corrigida do ventrículo direito (FEVDc) foi calculada dividindo-se o fluxo pulmonar ajustado (fluxo pulmonar anterógrado menos o fluxo regurgitante) pelo VDF do VD: [FEVDc = fluxo pulmonar líquido (fluxo pulmonar anterógrado − fluxo regurgitante) / VDF do VD].^13,14^ A fração de ejeção foi determinada através de duas sequências durante a imagem CMR. Em primeiro lugar, a sequência de “contraste de fase” permite medições diretas de fluxo no tronco pulmonar. Ao avaliar os fluxos anterógrados e retrógrados nesta região, torna-se possível calcular a diferença entre esses fluxos por ciclo cardíaco. Essa diferença representa o volume real de sangue ejetado pelo VD. Além disso, as medidas do volume cavitário do VD são obtidas por meio de cine-RM com cortes contíguos no eixo curto.^13^

### Ecocardiografia

A avaliação dos pacientes incluiu a avaliação das dimensões, volumes e função do VD seguindo as diretrizes recomendadas pela American Society of Echocardiography.^15,16^ Foi utilizado um sistema Phillips iE33 (Philips Medical Systems, Andover, MA, EUA), equipado com um transdutor multifrequencial (3,5 MHz e 5,0 MHz) e um transdutor de matriz X3 (1-3 MHz) para permitir a aquisição em tempo real de dados volumétricos usando a técnica de imagem de volume total. As imagens de repouso foram obtidas com monitorização eletrocardiográfica contínua, gravadas digitalmente e armazenadas no disco rígido e em CDs do aparelho em formato de arquivo CD/DVD (dados brutos) para análise subsequente. A deformação miocárdica off-line e a análise da reconstrução tridimensional foram realizadas utilizando software dedicado (TOMTEC Imaging Systems, Unterschleissheim, Alemanha, versão 4.3). Os valores resultantes representaram a avaliação média derivada de 3 ciclos cardíacos consecutivos.

O objetivo da avaliação das lesões residuais foi identificar e quantificar potenciais anormalidades na via de saída do VD, estenose pulmonar supravalvar, regurgitação pulmonar, regurgitação tricúspide e comunicação interventricular residual. A regurgitação pulmonar foi avaliada por Doppler com mapeamento de fluxo a cores nos planos paraesternal transversal, paraesternal esquerdo alto e supraesternal transversal. Isso permitiu a identificação de padrões de fluxo diastólico reverso na via de saída do VD, no tronco pulmonar e nas artérias pulmonares. A regurgitação pulmonar leve foi definida pela presença de fluxo diastólico correspondente ao refluxo pulmonar detectado no mapeamento de fluxo a cores próximo à valva pulmonar, juntamente com padrão Doppler de onda contínua mostrando fluxo holodiastólico. A regurgitação pulmonar moderada foi determinada pela detecção de fluxo diastólico no tronco pulmonar. Em contrapartida, a regurgitação pulmonar importante foi caracterizada pela presença de fluxo diastólico reverso nas artérias pulmonares, acompanhado de padrão protodiastólico na análise Doppler de onda contínua e intervalo de pressão inferior a 100 ms.^17,18^

### Análise estatística

O teste de Kolmogorov-Smirnov avaliou a normalidade das variáveis contínuas. Os dados são apresentados como média/desvio padrão ou mediana/intervalo interquartil, dependendo da distribuição das variáveis. Variáveis categóricas são expressas como números/porcentagens. A análise descritiva avaliou dados demográficos e cirúrgicos. Testes t pareados avaliaram alterações nos volumes e funções ventriculares antes e depois do IVP. A análise atuarial de Kaplan-Meier avaliou a probabilidade de sobrevivência livre de gradiente de pico superior a 60 mmHg e a necessidade de troca de prótese pulmonar, comparando diferentes tamanhos de prótese. A análise estatística utilizou o SPSS, versão 23, com significância estatística estabelecida em p < 0,05.

## Resultados

O estudo incluiu um total de 56 pacientes, 71% do sexo masculino, em pós-operatório tardio de correção de TF com regurgitação valvar pulmonar moderada a grave. Os dados demográficos e cirúrgicos são apresentados na Tabela 1.

**Tabela 1:** – Características demográficas dos pacientes

Variáveis	N = 56
Idade na avaliação (anos)	29 (24-34)
Sexo (masculino: feminino)	40:16
Idade na correção da TF (anos)	3 (2-5)
Tempo de acompanhamento após correção da TF (anos)	24 (22-30)
Idade no IVP (anos)	21 (18-25)
Tempo de acompanhamento após IVP (anos)	7 (6-9)
Tempo entre correção da TF e IVP (anos)	17 (14-21)

*IVP: implante valvar pulmonar; TF: tetralogia de Fallot.*

**Figura 1:**
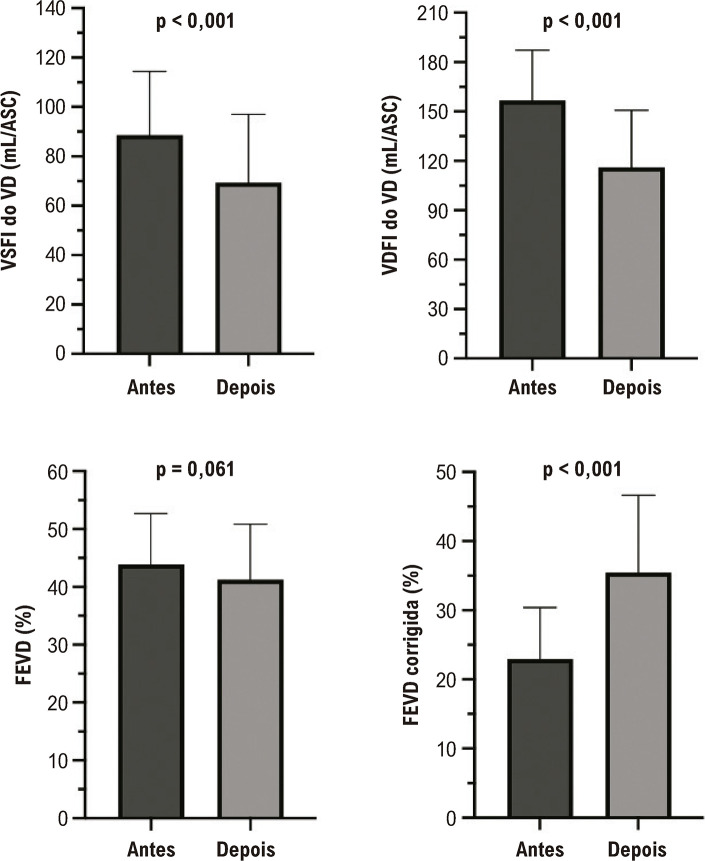
– Avaliação das alterações dos volumes e da fração de ejeção do ventrículo direito após implante valvar pulmonar. ASC: área de superfície corporal; FEVD: fração de ejeção do ventrículo direito; VD: ventrículo direito; VDFI: volume diastólico final indexado; VSFI: volume sistólico final indexado. Teste t pareado; significância estatística p < 0,05.

**Figura 2:**
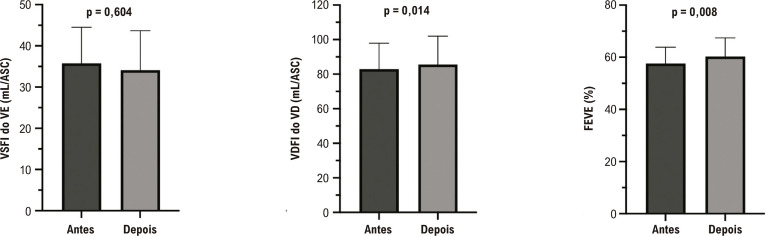
– Avaliação das alterações dos volumes e da fração de ejeção do ventrículo esquerdo após implante valvar pulmonar. ASC: área de superfície corporal; FEVE: fração de ejeção do ventrículo esquerdo; VDFI: volume diastólico final indexado; VE: ventrículo esquerdo; VSFI: volume sistólico final indexado. Teste t pareado; significância estatística p < 0,05.

Após o IVP, houve uma melhora significativa no VSFI do VD e no VDFI do VD. Embora não tenha havido alteração na FEVD medida pelo método de Simpson, observou-se aumento significativo quando utilizado o método da fração de ejeção corrigida, considerando o volume de regurgitação (Figura 1). Além disso, houve melhora no VSFI do VE, no VDFI do VE e na FEVE (Figura 2).

Observamos um aumento progressivo e significativo do gradiente de pico da prótese, provavelmente devido à degeneração. Foram avaliados 14 pacientes (25%) com gradiente de pico na prótese pulmonar > 60 mmHg e indicação de troca valvar.

**Figura 3:**
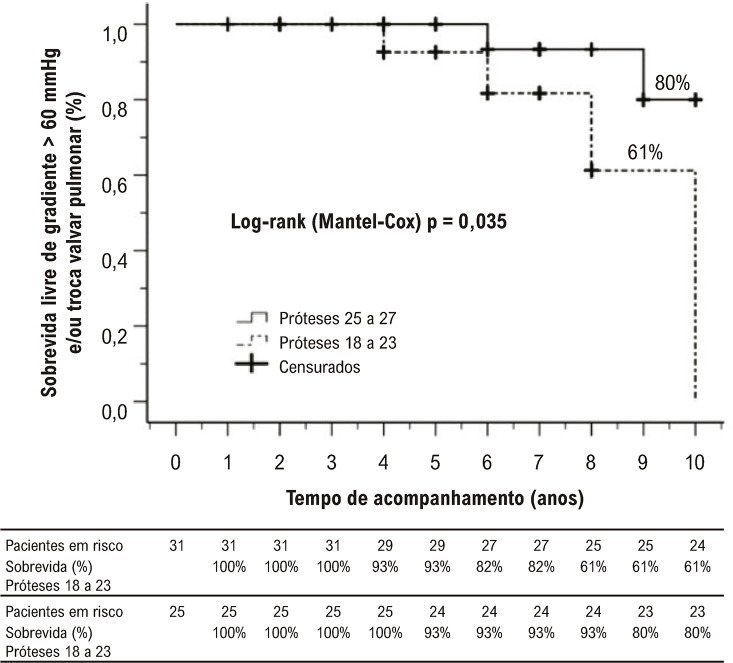
– Análise atuarial de Kaplan-Meier de sobrevida livre de gradiente de pico superior a 60 mmHg e/ou necessidade de troca valvar pulmonar.

A Figura 3 demonstra pior desfecho em 10 anos para pacientes que receberam próteses menores (tamanhos 19 a 23) em comparação com aqueles que receberam próteses maiores (tamanhos 25 e 27). Especificamente, próteses menores foram associadas a um risco 4,3 vezes maior de desenvolver um gradiente de pressão superior a 60 mmHg quando comparadas com próteses de tamanhos maiores (p = 0,029; intervalo de confiança: 1,18 a 17,8).

## Discussão

Os resultados do estudo ressaltam o impacto benéfico do IVP na função ventricular, conforme evidenciado por uma diminuição notável no VSFI e no VDFI do VD. Os sinais de remodelamento do VD estão de acordo com os achados de Geva et al.,^19^ que realizaram um estudo randomizado comparando IVP com e sem cirurgia de remodelamento do VD e observaram melhorias significativas nessas medidas no grupo IVP.

Apesar da controvérsia contínua em torno da potencial melhora da FEVD após o implante de prótese pulmonar, há evidências convincentes que apoiam a função melhorada do VD quando se utiliza a FEVD corrigida, conforme proposto por Heng et al.^14^ Essa métrica leva em consideração a presença de regurgitação pulmonar. Com o emprego dessas técnicas, torna-se possível avaliar o impacto do implante da prótese pulmonar na função do VD e monitorar as alterações ao longo do tempo. Embora a controvérsia permaneça, esses métodos fornecem informações valiosas sobre as potenciais melhorias observadas na função do VD após tais intervenções. Vliegen et al.^13^ também relataram melhorias na função do VD após IVP em pacientes com TF.

Os desfechos favoráveis observados após IVP ressaltam a importância da adoção de uma abordagem abrangente no manejo de pacientes com TF. Esta abordagem envolve vários aspectos, incluindo estratificação de risco completa, seleção de pacientes e intervenções personalizadas. Ao decidir sobre o momento ideal para IVP no pós-operatório tardio de correção de TF, vários fatores devem ser levados em consideração, como VDFI do VD, FEVD, presença de aneurisma na via de saída do VD, anormalidades eletrocardiográficas e sintomas clínicos. Ao considerar esses fatores, uma abordagem mais individualizada e personalizada pode ser empregada para garantir os melhores desfechos possíveis para pacientes com TF submetidos a IVP. Oosterhof et al.^20^ sugeriram que os limiares pré-operatórios, incluindo o VDFI do VD e a FEVD, podem ajudar a orientar o momento apropriado para a intervenção. Esses estudos destacam a importância de adaptar o momento do IVP às características específicas e ao perfil de risco de cada paciente. Em nosso estudo, realizamos uma avaliação personalizada, considerando fatores como VDFI, FEVD, capacidade funcional e sintomas clínicos. Apesar dos efeitos positivos do IVP na função do VD e nos desfechos clínicos em pacientes com TF corrigida, alguns indivíduos ainda apresentam fadiga e capacidade funcional reduzida mesmo após serem submetidos a IVP.^21-24^ Esses achados sugerem que o IVP por si só pode não restaurar completamente a capacidade funcional normal em todos os pacientes com TF. Consequentemente, mais pesquisas são necessárias para compreender melhor os fatores que contribuem para esses sintomas persistentes e para explorar intervenções adicionais que possam ser benéficas. Infelizmente, nosso estudo não avaliou a capacidade funcional dos pacientes antes e depois do IVP, o que representa uma limitação da nossa pesquisa. No entanto, os resultados destacam a importância de considerar abordagens individualizadas na determinação do momento ideal para o IVP e a necessidade de investigações adicionais para melhorar os desfechos e abordar os sintomas persistentes em pacientes com TF.

Embora o foco dos desfechos de IVP tenha sido historicamente centrado no VD, também ocorre remodelamento do VE. Estudos anteriores relacionaram regurgitação pulmonar significativa com disfunção sistólica do VE e destacaram a importância da função do VE no prognóstico da TF corrigida. Nossos achados mostraram que o IVP pode levar à melhora da função do VE, semelhante a outras publicações.^14,20^ A melhora observada na função do VE pode ser parcialmente explicada pela atenuação do fenômeno de Bernheim, onde as alterações do VD impactam negativamente a função do VE.^25^ A instalação de uma válvula pulmonar competente melhora a eficiência do VD e aumenta a pré-carga do VE, aumentando o volume diastólico final e a função do VE.

O acompanhamento e a vigilância a longo prazo após IVP são aspectos essenciais do cuidado de pacientes com TF. As características estruturais e funcionais da válvula bioprotética são fundamentais para um ótimo desempenho após IVP. Caldarone et al.,^26^em uma análise retrospectiva de 945 operações em 726 pacientes submetidos a IVP, mostraram uma interação significativa entre idade, tipo de válvula e tamanho na longevidade da prótese valvar pulmonar. As válvulas bioprotéticas podem ser construídas de pericárdio bovino ou válvulas porcinas com ou sem *stents* e com diversas propriedades geométricas. As válvulas bioprotéticas porcinas consistem em 3 folhetos da válvula aórtica porcina reticulados com glutaraldeído e montados em um *stent* de suporte metálico ou polimérico. As válvulas pericárdicas são fabricadas a partir de lâminas de pericárdio bovino montadas dentro ou fora de um *stent* de suporte. Em nossa prática, a maioria das trocas valvares comumente utilizadas em unidades de saúde em todo o mundo estão prontamente disponíveis.^27^ Porém, considerando o alto custo associado às próteses comumente citadas na literatura, optamos por utilizar próteses de fabricação nacional na nossa instituição devido aos seus preços mais acessíveis. Embora a durabilidade dessas próteses bovinas de fabricação nacional na posição pulmonar careça de dados específicos da literatura, e os estudos comparativos com outras opções valvares mundialmente reconhecidas sejam limitados, continuamos monitorando de perto seu desempenho e desfechos. Nosso estudo revelou um aumento progressivo no gradiente de pico da válvula pulmonar ao longo do tempo, o que pode ser atribuído à degeneração valvar. Esse aumento atinge seu grau máximo aproximadamente 6 anos após a alta hospitalar. Esses achados enfatizam a importância do monitoramento contínuo para avaliar a durabilidade a longo prazo das válvulas implantadas e detectar possíveis complicações. Em uma revisão retrospectiva de 227 pacientes com TF submetidos à troca valvar pulmonar usando bioprótese com *stent* no Children’s Hospital Boston entre 1994 e 2009, Chen et al.^28^ não identificaram o tipo de prótese como preditor de deterioração estrutural da valva nesta população. Porém, não utilizaram próteses de pericárdio bovino. No entanto, reconheceram que a idade mais jovem no momento da substituição da válvula pulmonar e o sobredimensionamento da válvula em pacientes com menos de 20 anos de idade no momento da troca da válvula pulmonar eram preditores significativos de deterioração estrutural da válvula.^28^ A vigilância a longo prazo deve incluir a avaliação regular da função da válvula, da hemodinâmica e dos sintomas do paciente para garantir uma intervenção oportuna e otimizar os desfechos. Vliegen et al.^13^ enfatizaram a importância de avaliar a função valvar ao longo do tempo para monitorar potencial degeneração por meio de ressonância magnética.

O dimensionamento da prótese é outro fator que merece atenção nos procedimentos de IVP. Nossa análise revelou uma associação preocupante entre próteses menores (tamanho 19 a tamanho 23) e um maior risco de degeneração ao longo do tempo, necessitando de substituição valvar mais precoce do que as próteses maiores (tamanho 25 e tamanho 27). Frigiola et al.^29^ verificaram que pacientes que receberam próteses menores apresentavam maior risco de degeneração e potencial necessidade de nova troca do que aqueles que receberam próteses maiores. O maior risco de deterioração valvar no grupo com próteses de menor tamanho levanta preocupações quanto à seleção adequada da prótese e seu impacto nos desfechos a longo prazo. Mais pesquisas são necessárias para refinar estratégias de dimensionamento e otimizar a durabilidade a longo prazo.

Em resumo, IVP afetou positivamente a função do VD e os desfechos clínicos em pacientes com TF. No entanto, desafios como a fadiga persistente e a redução da capacidade funcional permanecem. A estratificação abrangente do risco, a seleção dos pacientes e as intervenções personalizadas são cruciais para um manejo eficaz. O acompanhamento e a vigilância a longo prazo são necessários para avaliar a durabilidade das válvulas implantadas e detectar potenciais complicações. Compreender o impacto do IVP em pacientes com TF pode orientar as decisões de tratamento e melhorar o seu prognóstico a longo prazo.

## Conclusão

Conforme esperado, o IVP demonstrou melhorias nos volumes e na função do VD. O acompanhamento e a vigilância a longo prazo são cruciais para avaliar a durabilidade da prótese e detectar potenciais complicações. O dimensionamento adequado das próteses é essencial para obter desfechos ideais. Esses achados destacam a importância do IVP no manejo de pacientes com TF, enfatizando a necessidade de monitoramento cuidadoso e a seleção de próteses adequadas para garantir sucesso em longo prazo.

### Limitações do estudo

O presente estudo possui várias limitações potenciais. Em primeiro lugar, o seu desenho retrospectivo e unicêntrico introduz potenciais vieses, uma vez que se baseia em dados históricos de apenas uma instituição médica. Essa abordagem pode não capturar com precisão a evolução das técnicas cirúrgicas e do conhecimento médico, potencialmente tornando os achados menos aplicáveis às práticas atuais. A variabilidade nos estágios em que os pacientes foram encaminhados para cirurgia de IVP, especialmente dadas as mudanças nas diretrizes clínicas e nos critérios de tomada de decisão de 2011 até o presente, sugere ainda que os pacientes estudados podem não representar totalmente a atual população de pacientes submetidos a IVP.^10,11,30,31^ As restrições econômicas no sistema de saúde que influenciaram a escolha dos dispositivos protéticos também representam uma limitação significativa. Essas restrições podem ter impedido a adoção de tecnologias protéticas mais novas e potencialmente mais eficazes, não demonstrando assim totalmente os avanços nos desfechos de IVP. Finalmente, nosso estudo não incluiu teste de exercício cardiopulmonar para avaliar alterações na capacidade funcional após o IVP, o que restringe nossa capacidade de compreender de forma abrangente o significado clínico das melhorias cardíacas observadas. Isso torna difícil medir com precisão como o IVP afeta os níveis de atividade física e o bem-estar geral dos pacientes. Essas limitações realçam a importância de mais pesquisas em ambientes mais diversos e contemporâneos para validar e expandir os achados do presente estudo.
