# Supplementary material for: Efeitos de Longo Prazo do Implante Valvar Pulmonar e Evolução da Prótese em Pacientes com Tetralogia de Fallot Corrigida
Source: Arq Bras Cardiol. 2024 Jul 10;121(7):e20230585. [Article in Portuguese] doi: 10.36660/abc.20230585 (PMC12087634; doi:10.36660/abc.20230585)
Supplement: Translation

# Translation: Long-Term Effects of Pulmonary Valve Implantation and Prosthesis Evolution in Patients with Repaired Tetralogy of Fallot

**DOI:** 10.36660/abc.20230585i

**Authors:** Luiz Fernando Caneo, Aida Luiza Ribeiro Turquetto, Matheus Negri Boschiero, Luciana Patrick Amato, Walther Yoshiharu Ishikawa, Fabiana Padilha Hodas, Melissa Ganeko Ligeiro, Daniela Regina Agostinho, Leonardo Augusto Miana, Carla Tanamati, Rilvani Cavalcante Gonçalves, Juliano Gomes Penha, Maria Raquel Brigoni Massoti, Marcelo Biscegli Jatene, Fabio Biscegli Jatene

**Affiliations:** 1 Brazil Instituto do Coração do Hospital das Clínicas da Faculdade de Medicina da Universidade de São Paulo, São Paulo, SP – Brazil; 2 Brazil Universidade São Francisco Faculdade de Medicina, Bragança Paulista, SP – Brazil

**Keywords:** Tetralogy of Fallot, Right Ventricular Function, Biomarkers, Genetic Risk Score

## Abstract

**Background:**

Pulmonary valve regurgitation is a significant long-term complication in patients with tetralogy of Fallot (TOF).

**Objective:**

This study aims to investigate the effects of pulmonary valve implantation (PVI) on the anatomy and function of the right ventricle (RV) and the long-term evolution of the implanted prosthesis in the pulmonary position.

**Methods:**

A single-center retrospective cohort analysis was performed in 56 consecutive patients with TOF who underwent PVI. The study included patients of both sexes, aged ≥ 12 years, and involved assessing clinical and surgical data, pre- and post-operative cardiovascular magnetic resonance imaging, and echocardiogram data more than 1 year after PVI.

**Results:**

After PVI, there was a significant decrease in RV end-systolic volume indexed by body surface area (BSA), from 89 mL/BSA to 69 mL/BSA (p < 0.001) and indexed RV end-diastolic volume, from 157 mL/BSA to 116 mL/BSA (p < 0.001). Moreover, there was an increase in corrected RV ejection fraction [[RVEFc = net pulmonary flow (pulmonary forward flow − regurgitant flow) / RV end-diastolic volume] from 23% to 35% (p < 0.001) and left ventricular ejection fraction from 58% to 60% (p = 0.008).] from 23% to 35% (p < 0.001) and left ventricular ejection fraction from 58% to 60% (p = 0.008). However, a progressive increase in the peak pulmonary valve gradient was observed over time, with 25% of patients experiencing a gradient exceeding 60 mmHg. Smaller prostheses (sizes 19 to 23) were associated with a 4.3-fold higher risk of a gradient > 60 mmHg compared to larger prostheses (sizes 25 to 27; p = 0.029; confidence interval: 1.18 to 17.8).

**Conclusion:**

As expected, PVI demonstrated improvements in RV volumes and function. Long-term follow-up and surveillance are crucial for assessing the durability of the prosthesis and detecting potential complications. Proper sizing of prostheses is essential for improved prosthesis longevity.

## Introduction

Pulmonary valve regurgitation is a common long-term complication observed in patients who have undergone surgical repair for tetralogy of Fallot (TOF). This condition leads to various detrimental effects such as progressive enlargement and impaired function of the right ventricle (RV), irregular heart rhythms, heart failure, reduced exercise capacity, and an elevated risk of sudden and premature death.^1,2^

Pulmonary valve implantation (PVI) has emerged as a promising treatment approach to address these issues. However, determining the optimal timing for PVI presents a challenge, requiring precise risk assessment.^3^ It is important to note that, even after achieving initial improvements in RV size and function through PVI, the implanted valve may still deteriorate over time.^4^ Optimal timing of PVI remains challenging, as there needs to be a balance between intervening too early and risking complications, such as endocarditis or repeated PVI procedures, due to the limited durability of prosthetic valves. On the other hand, intervening too late may result in limited potential for RV functional recovery, leading to late-onset arrhythmias and heart failure.^5,6^ Furthermore, regardless of the prosthesis size, reintervention rates vary according to the type of valve implanted.^7^ The absence of uniformity in valve selection protocols within and among healthcare facilities, driven by surgeon preference or the chronological period of implantation, interferes with the systematic analysis of published outcomes.^8,9^ In contrast to developed countries, not all commercially used prostheses from studies are available domestically, mainly in the public health sector, due to their elevated costs.

This study aims to investigate the effects of PVI on the anatomy and function of the RV and the long-term evolution of the implanted prosthesis in the pulmonary position.

## Methods

This is single-center retrospective cohort study performed in 56 consecutive patients in the late postoperative repair of TOF who underwent PVI due to moderate to severe pulmonary regurgitation between 2011 and 2018 at our institution (Central Illustration). The institution’s Ethics Committee on Human Research approved the study under CAAE 50224615.7.0000.0068.

### Inclusion criteria

This study included patients who had undergone TOF repair and subsequently received PVI when symptomatic or when asymptomatic in those with severe pulmonary regurgitation with at least one of the following criteria: progressive objective reduction in exercise capacity, progressive RV dilation, progressive RV systolic dysfunction, progressive tricuspid regurgitation (at least moderate) or sustained atrial/ventricular arrhythmias.^10,11^ Individualized recommendations for PVI were made after multidisciplinary reviews. Informed consent from the patients to participate in the study was required. The study included patients of both sexes, aged 12 years or older, with follow-up time of more than 1 year after PVI, who accepted to participate in the study and signed informed consent.

### Surgical details

Surgery was performed using cardiopulmonary bypass and a beating heart when feasible. A transannular patch was used in 55% of the patients to repair the TOF, which was substituted or added in 81% during PVI due to the calcification of the original bovine pericardium patch. A domestically manufactured bovine pericardial prosthesis (Braile Biomedica, Sao Paulo, Brazil) was implanted in all patients, with a size ranging from 19 to 27 mm.

### Image evaluation

We collected and analyzed data obtained from cardiovascular magnetic resonance (CMR) imaging and echocardiography. CMR examinations were conducted at two time points: before the surgery (preoperative) and more than one year after PVI. The purpose of CMR analysis was to evaluate the indexed end-systolic and diastolic volumes (ESVi and EDVi) of the RV, right ventricular ejection fraction (RVEF), and left ventricular ejection fraction (LVEF). We used Kaplan-Meier curves to analyze the survival free from peak gradient greater than 60 mmHg and/or indication for pulmonary valve replacement according to the prosthesis size used in the surgery. The follow-up time was calculated using the date of the last echocardiogram obtained in the postoperative follow-up or before the pulmonary valve replacement subtracted from the date of the PVI.

### Cardiovascular magnetic resonance

All patients had CMR examinations on a 1.5T magnetic resonance imaging (MRI) system (Philips Achieva scanner, Netherlands). The ventricular function was evaluated with cine-MRI with SSFP (steady-state free precession) sequence. Volumes and ventricular function were measured by the Simpson’s method on short-axis images and were indexed to body surface area. Stroke volume was calculated by deducting the ESVi from the EDVi. Pulmonary flow and regurgitation were measured with phase contrast sequences. Contrast-enhanced magnetic resonance angiography was used to visualize the pulmonary vascular tree and connections, and late gadolinium enhancement was used to detect myocardial fibrosis (by thresholding technique).^12^ The pulmonary regurgitant fraction was calculated from phase-velocity mapping in a plane transecting the main pulmonary trunk. Corrected right ventricular ejection fraction (RVEFc) was calculated by dividing the net pulmonary flow (pulmonary forward flow minus the regurgitant flow) by the RVEDV: [RVEFc = net pulmonary flow (pulmonary forward flow − regurgitant flow) / RVEDV].^13,14^ The ejection fraction is determined through two sequences during CMR imaging. Firstly, the “phase contrast” sequence enables direct flow measurements in the pulmonary trunk. By assessing both anterograde and retrograde flows in this region, it becomes possible to calculate the difference between these flows on a per-cardiac cycle basis. This difference represents the actual volume of blood ejected by the RV. Additionally, cavitary volume measurements of the RV are obtained using cine-MRI with contiguous short-axis slices.^13^

### Echocardiography

Patient evaluation included the assessment of RV dimensions, volumes, and function following the guidelines recommended by the American Society of Echocardiography.^15,16^ A Phillips iE33 system (Philips Medical Systems, Andover, MA, USA) was utilized, equipped with a multifrequency transducer (3.5 MHz and 5.0 MHz) and an X3 matrix transducer (1-3 MHz) to enable real-time acquisition of volumetric data using the full-volume imaging technique. Resting images were obtained with continuous electrocardiographic monitoring, recorded digitally, and stored on the device’s hard disk and CDs in CD/DVD file format (raw data) for subsequent analysis. Offline myocardial deformation and three-dimensional reconstruction analysis were performed using dedicated software (TOMTEC Imaging Systems, Unterschleissheim, Germany, version 4.3). The resulting values represented the average evaluation derived from three consecutive cardiac cycles.

The objective of assessing residual lesions was to identify and quantify potential abnormalities in the RV outflow tract, supra-valvular pulmonary stenosis, pulmonary regurgitation, tricuspid regurgitation, and residual interventricular communication. Pulmonary regurgitation was evaluated using Doppler imaging with color flow mapping in the transverse parasternal, high left parasternal, and transverse suprasternal planes. This allowed for identification of diastolic reverse flow patterns in the RV outflow tract, pulmonary trunk, and pulmonary arteries. Mild pulmonary regurgitation was defined by the presence of diastolic flow corresponding to pulmonary reflux detected in the color flow mapping near the pulmonary valve, along with a continuous-wave Doppler pattern showing holodiastolic flow. Moderate pulmonary regurgitation was determined by detecting diastolic flow in the pulmonary trunk. In contrast, significant pulmonary regurgitation was characterized by the presence of diastolic reverse flow in the pulmonary arteries, accompanied by a proto-diastolic pattern in the continuous-wave Doppler analysis and a pressure half-time less than 100 ms.^17,18^

### Statistical analysis

The Kolmogorov-Smirnov test assessed the normality of continuous variables. Data are presented as mean/standard deviation or median/interquartile range, depending on variable distribution. Categorical variables are expressed as numbers/percentages. Descriptive analysis examined demographic and surgical data. Paired t tests evaluated changes in ventricular volumes and functions before and after PVI. Kaplan-Meier actuarial analysis assessed the probability of survival free from a peak gradient exceeding 60 mmHg and the need for pulmonary prosthesis replacement, comparing different prosthesis sizes. Statistical analysis used SPSS, version 23, with statistical significance set at p < 0.05.

## Results

The study included a total of 56 patients, 71% male, in the late postoperative period of TOF correction with moderate to severe pulmonary valve regurgitation. Demographic and surgical data are presented in Table 1.

**Table 1:** – Demographic characteristics of patients

Variáveis	N = 56
Age at evaluation (years)	29 (24-34)
Sex (male: female)	40:16
Age at TOF repair (years)	3 (2-5)
Follow-up time after TOF repair (years)	24 (22-30)
Age at PVI (years)	21 (18-25)
Follow-up time after PVI (years)	7 (6-9)
Time between TOF repair and PVI (years)	17 (14-21)

*PVI: pulmonary valve implantation; TOF: tetralogy of Fallot.*

Following PVI, there was a significant improvement in RV ESVi and RV EDVi. Although there was no change in the RVEF measured by Simpson’s method, a significant increase was observed when the corrected ejection fraction method, considering the regurgitation volume, was utilized. (Figure 1). Additionally, there was an improvement in LV ESVi, LV EDVi, and LVEF (Figure 2).

**Figure 1:** – Assessment of RV volume and ejection fraction changes following pulmonary valve implantation. BSA: body surface area; EDVi: indexed end-diastolic volume; EF: ejection fraction; ESVi: indexed end-systolic volume; RV: right ventricle. Paired t Test; statistical significance p < 0.05.

**Figure 2:** – Assessment of left ventricular volume and ejection fraction changes following pulmonary valve implantation. BSA: body surface area; EDVi: indexed end-diastolic volume; EF: ejection fraction; ESVi: indexed end-systolic volume; LV: left ventricular. Paired t Test; statistical significance p < 0.05.

A progressive and significant increase in the peak gradient of the prosthesis was observed, probably due to degeneration. Fourteen patients (25%) were evaluated with a peak gradient in the pulmonary prosthesis > 60 mmHg and an indication for valve re-replacement.

Figure 3 demonstrates a worse outcome over 10 years for patients implanted with smaller prostheses (sizes 19 to 23) than individuals who received larger prostheses (sizes 25 and 27). Specifically, smaller prostheses were linked to a 4.3 times greater risk of developing a pressure gradient exceeding 60 mmHg when contrasted with the larger prosthetic sizes (p = 0.029; confidence interval: 1.18 to 17.8).

**Figure 3:** – Kaplan-Meier actuarial analysis of freedom from peak gradient exceeding 60 mmHg and/or the need for pulmonary valve replacement.

## Discussion

The study findings underscore the beneficial impact of PVI on ventricular function, as evidenced by a notable decrease in RV ESVi and RV EDVi. The signs of RV remodeling align with the findings of Geva et al.,^19^ who conducted a randomized trial comparing PVI with and without RV remodeling surgery and observed significant improvements in these measures in the PVI group.

Despite ongoing controversy surrounding the potential improvement of RVEF after pulmonary prosthesis implantation, there is compelling evidence supporting enhanced RV function when using the RVEFc, as proposed by Heng et al.^14^ This metric takes into account the presence of pulmonary regurgitation. By employing these techniques, it becomes possible to assess the impact of pulmonary prosthesis implantation on RV function and monitor changes over time. While the controversy remains, these methods provide valuable insights into the potential improvements observed in RV function following such interventions.

The favorable outcomes observed after PVI underscore the importance of adopting a comprehensive approach to managing patients with TOF. This approach entails various aspects, including thorough risk stratification, patient selection, and personalized interventions. When deciding on the optimal timing for PVI in late postoperative TOF repair, several factors should be taken into consideration, such as RV EDVi, RVEF, the presence of aneurysm in the RV outflow tract, electrocardiographic abnormalities, and clinical symptoms. By considering these factors, a more individualized and tailored approach can be employed to ensure the best possible outcomes for TOF patients undergoing PVI. Oosterhof et al.^20^ suggested that preoperative thresholds, including RV EDVi and RVEF, can help guide the appropriate timing for intervention. These studies highlight the importance of tailoring the timing of PVI to each patient’s specific characteristics and risk profile. In our study, we conducted a personalized evaluation, considering factors such as EDVi, RVEF, functional capacity, and clinical symptoms. Despite the positive effects of PVI on RV function and clinical outcomes in patients with repaired TOF, some individuals still experience fatigue and reduced functional capacity even after undergoing PVI.^21-24^ These findings suggest that PVI alone may not fully restore normal functional capacity in all patients with TOF. As a result, further research is necessary to understand better the factors contributing to these persistent symptoms and to explore additional interventions that may be beneficial. Unfortunately, our study did not assess the functional capacity of patients before and after PVI, which represents a limitation of our research. Nonetheless, the findings underscore the importance of considering individualized approaches in determining the optimal timing for PVI and the need for additional investigations to improve outcomes and address lingering symptoms in patients with TOF.

Although the focus of PVI outcomes has historically centered on the RV, LV remodeling also occurs. Previous studies have linked significant pulmonary regurgitation with LV systolic dysfunction and highlighted the importance of LV function in the prognosis of repaired TOF. Our findings showed that PVI can lead improvement in LV function, similar to other publications.^14,20^ The observed improvement in LV function could be partly explained by mitigating of the Bernheim phenomenon, where RV alterations negatively impact LV function.^25^ Installing a competent pulmonary valve improves RV efficiency and increases LV preload, boosting LV end-diastolic volume and function.

Long-term follow-up and surveillance after PVI are essential aspects of TOF patient care. Structural and functional characteristics of the bioprosthetic valve are fundamental for optimal performance after PVI. Calderone et al.,^26^in a retrospective analysis of 945 operations in 726 patients undergoing PVI, showed a significant interaction between age, valve type, and size on prosthetic pulmonary valve longevity. Bioprosthetic valves can be constructed of bovine pericardium or porcine valves with or without stents and with various geometric properties. Porcine bioprosthetic valves consist of three porcine aortic valve leaflets cross-linked with glutaraldehyde and mounted on a metallic or polymer supporting stent. Pericardial valves are manufactured from sheets of bovine pericardium mounted inside or outside a supporting stent. In our practice, the majority of valve substitutes commonly used in healthcare facilities worldwide are readily available.^27^ However, considering the high cost associated with the prostheses commonly referenced in the literature, we have opted to utilize domestically manufactured prostheses in our institution due to their more affordable pricing. While the durability of these domestically manufactured bovine prostheses in the pulmonary position lacks specific literature data, and comparative studies with other globally recognized valve options are limited, we continue closely monitoring their performance and outcomes. Our study revealed a progressive increase in the peak pulmonary valve gradient over time, which can be attributed to valve degeneration. This increase reaches its maximum degree approximately 6 years after hospital discharge. These findings emphasize the importance of ongoing monitoring to assess the long-term durability of the implanted valves and detect potential complications. In a retrospective review of 227 patients with TOF who underwent stented bioprosthetic pulmonary valve replacement at Children’s Hospital Boston between 1994 and 2009, Chen et al.^28^ did not identify the type of prosthesis as a predictor of structural valve deterioration in this population. However, they did not use the bovine pericardial prosthesis. Nonetheless, they recognized that younger age at the time of pulmonary valve replacement and valve oversizing in patients under 20 years of age at the time of pulmonary valve replacement were significant predictors of structural valve deterioration.^28^ Long-term surveillance should include regular assessment of valve function, hemodynamics, and patient symptoms to ensure timely intervention and optimize outcomes. Vliegen et al.^13^ emphasized the importance of assessing valve function over time to monitor potential degeneration using MRI.

Prosthesis sizing is another factor that warrants attention in PVI procedures. Our analysis revealed a concerning association between smaller prostheses (size 19 to size 23) and a higher risk of degeneration over time, necessitating earlier valve re-replacement than larger prostheses (size 25 and size 27). Frigiola et al.^29^ found that patients who received smaller prostheses had a higher risk of degeneration and the potential need for re-replacement than those who received larger prostheses. The higher risk of valve deterioration in the group with smaller-size prostheses raises concerns regarding appropriate prosthesis selection and its impact on long-term outcomes. Further research is needed to refine sizing strategies and optimize long-term durability.

In summary, PVI positively affected RV function and clinical outcomes in TOF patients. However, challenges such as persistent fatigue and reduced functional capacity remain. Comprehensive risk stratification, patient selection, and personalized interventions are crucial for effective management. Long-term follow-up and surveillance are necessary to assess the durability of implanted valves and detect potential complications. Understanding the impact of PVI on patients with TOF can guide treatment decisions and enhance their long-term prognosis.

## Conclusion

As expected, PVI demonstrated improvements in RV volumes and function. Long-term follow-up and surveillance are crucial for assessing the durability of the prosthesis and detecting potential complications. Proper sizing of prostheses is essential for achieving optimal outcomes. These findings highlight the importance of PVI in managing patients with TOF, emphasizing the need for careful monitoring and appropriate prosthesis selection to ensure long-term success.

### Study limitations

This study has several potential limitations. Firstly, its retrospective, single-center design introduces potential biases, since it relies on historical data from only one medical institution. This approach may not accurately capture the evolution of surgical techniques and medical knowledge, potentially rendering the findings less applicable to current practices. The variability in the stages at which patients were referred for PVI surgery, especially given the changes in clinical guidelines and decision-making criteria from 2011 to the present, further suggests that the patients studied may not fully represent the current patient population undergoing PVI.^10,11,30,31^ Economic constraints within the healthcare system that influenced the choice of prosthetic devices also pose a significant limitation. These constraints may have prevented the adoption of newer, potentially more effective prosthetic technologies, thereby not fully showcasing the advancements in PVI outcomes. Finally, our study did not include cardiopulmonary exercise testing to evaluate functional capacity changes after PVI, which restricts our ability to comprehensively understand the clinical significance of the observed cardiac improvements. This makes it challenging to accurately measure how PVI affects patients’ physical activity levels and overall well-being. These limitations highlight the importance of further research in more diverse and contemporary settings to validate and expand upon these findings.
